# Sarcoid‐Like Reaction (SLR) Associated With Immune Checkpoint Inhibitors in Metastatic Renal Cell Carcinoma

**DOI:** 10.1002/iju5.70027

**Published:** 2025-04-14

**Authors:** Kanta Fukushima, Sota Mizukoshi, Tomohiro Ino, Takuya Ishida, Yumika Ito, Akira Okimura, Munehide Nakatsugawa, Takeshi Hashimoto

**Affiliations:** ^1^ Department of Urology Tokyo Medical University Hachioji Medical Center Tokyo Japan; ^2^ Department of Urology Tokyo Medical University Tokyo Japan; ^3^ Department of Diagnostic Pathology Tokyo Medical University Hachioji Medical Center Tokyo Japan

**Keywords:** immune checkpoint inhibitor, immune‐related adverse event, renal cell carcinoma, sarcoid‐like reaction

## Abstract

**Introduction:**

Immune checkpoint inhibitors (ICIs) are useful in the treatment of metastatic renal cell carcinoma. ICIs have been associated with a variety of immune‐related adverse events (irAEs), one of which is a sarcoid‐like reaction (SLR). SLR is often difficult to distinguish from cancer progression.

**Case Presentation:**

The patient is a 71‐year‐old woman. She underwent laparoscopic nephrectomy after 4 courses of ipilimumab + nivolumab therapy for clear cell renal cell carcinoma with femoral bone metastasis. In the pathological examination, in addition to tumor‐infiltrating lymphocytes, numerous non‐caseating epithelioid cell granulomas were found in the tumor. The occurrence of non‐caseating epithelioid cell granuloma in a patient who did not meet the diagnostic criteria for systemic sarcoidosis was considered SLR due to ICI.

**Conclusions:**

We report the first case of a patient who underwent surgery for metastatic renal cell carcinoma after treatment with ICI and who was pathologically diagnosed with SLR in the kidney.

**Summary**

- With immune checkpoint inhibitor therapy for malignant tumors, it is important to be aware of the possibility of sarcoidosis‐like reactions as immune‐related adverse events.
- Differentiation from cancer progression is important in determining future treatment strategies.

## 1 Introduction

Sarcoidosis is an example of non‐caseating granulomatous inflammation of unknown etiology characterized by multiorgan involvement. Non‐caseating granulomas resembling sarcoidosis may occasionally be seen in patients who do not meet the criteria for systemic sarcoidosis. Although these are called Sarcoid‐like reaction (SLR), but it is sometimes difficult to distinguish between the two [1, 2, 3]. SLR has been reported to be associated with a variety of malignancies and drugs [4, 5, 6, 7]. One of the most common agents is ICIs. ICIs are widely used as a breakthrough therapy in the treatment of metastatic renal cell carcinoma (RCC). Although ICIs have played a revolutionary role in cancer therapy by activating the immune system and enhancing anti‐tumor effects, ICIs have been associated with a variety of immune‐related adverse events (irAEs). Although guidelines from major cancer societies recognize SLR as a unique irAE caused by ICI, standardized treatment guidelines have not yet been established due to the paucity of literature on SLR and the rarity of the disease itself. This leaves the decision to treat or discontinue ICI to the discretion of the clinician [8, 9, 10, 11]. Importantly, this reaction may lead to the diagnosis of new metastatic disease and misdiagnosis of disease progression [12]. In this case report, we report our experience with a case of SLR associated with ICI in metastatic RCC with a literature review.

## 2 Case Presentation

**FIGURE 1:**
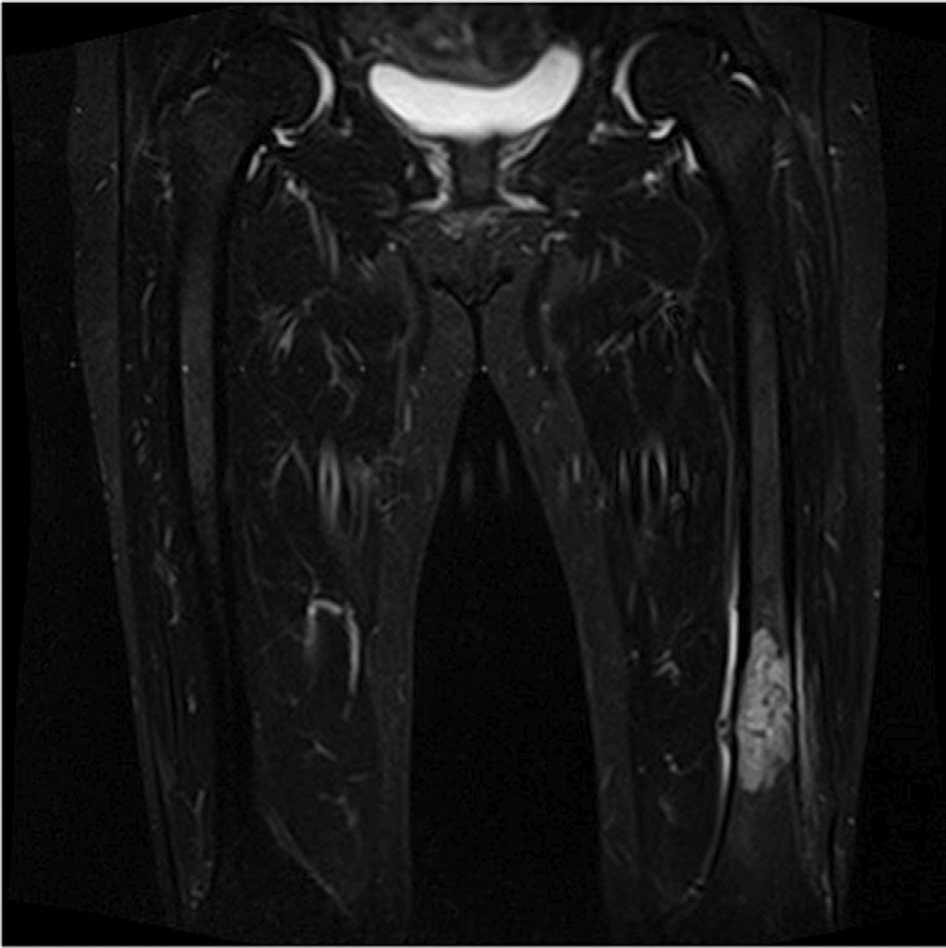
MRI showed findings of a malignant bone tumor of the left femur.

**FIGURE 2:**
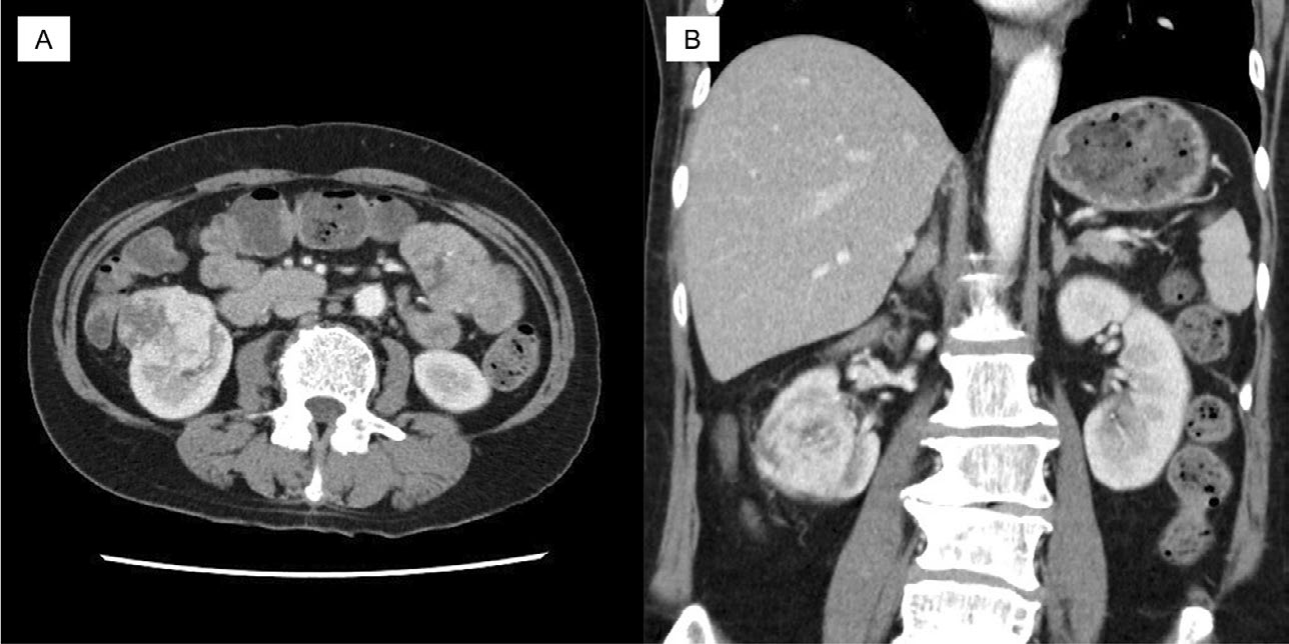
Contrast‐enhanced CT showed an approximately 5 cm renal tumor with contrast effect on the lower pole of the kidney. (A) Axial section (B) Coronal section.

A 71‐year‐old woman presented to an orthopedic hospital with a chief complaint of left femur pain. Her medical history includes diabetes mellitus. An MRI scan of the femur suspected a metastatic malignant bone tumor (Figure 1). An abdominal CT revealed a 49 mm RCC in the right kidney (Figure 2). Orthopedic surgery was performed to remove the malignant bone tumor, and all lesions were removed. Pathological examination showed metastatic clear cell carcinoma, and the patient was referred to our hospital for treatment. Based on IMDC criteria, this patient was classified as intermediate risk. Four courses of combination therapy with ipilimumab and nivolumab were administered; no irAE occurred.

**FIGURE 3:**
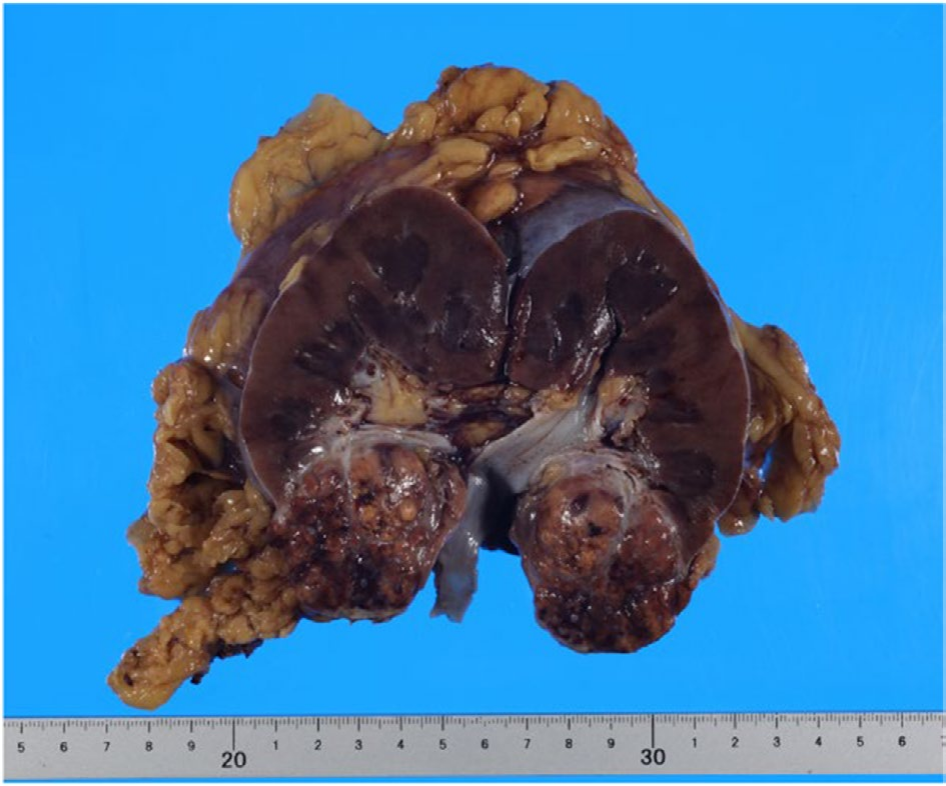
Gross findings of the resected tumor. A yellowish‐brown tumor was observed in the lower pole of the kidney.

**FIGURE 4:**
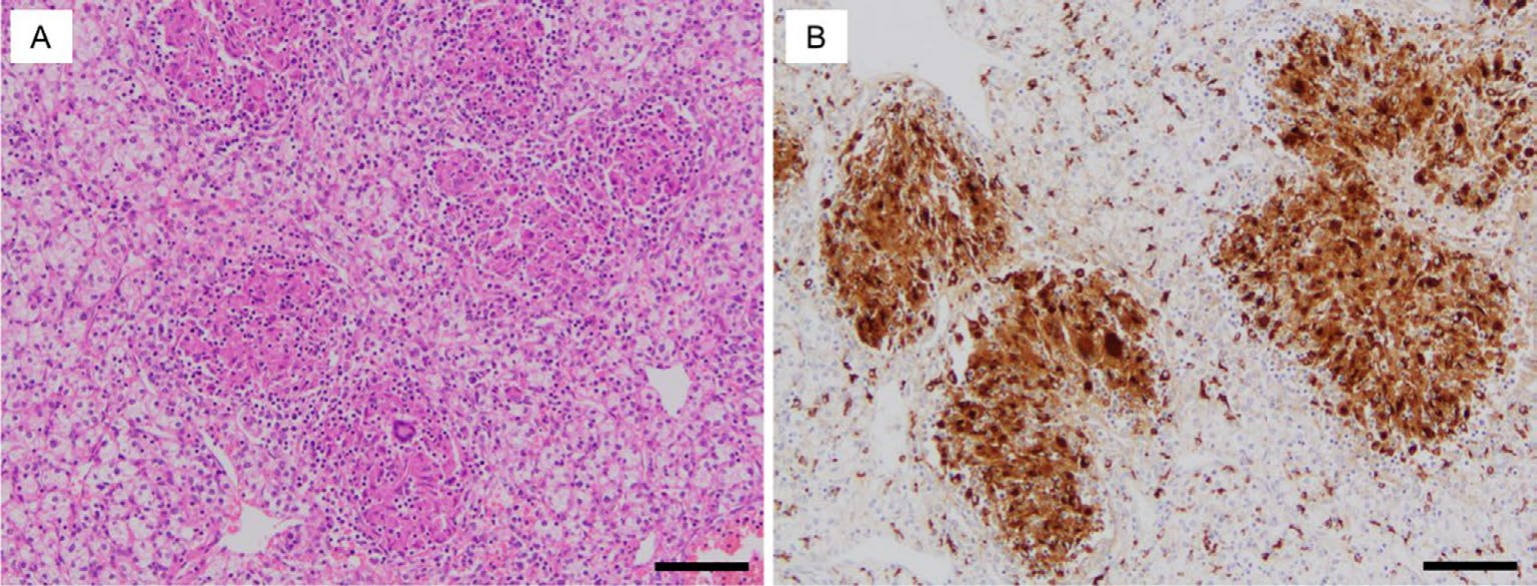
Histological findings of the resected tumor tissue. The tumor was analyzed by hematoxylin–eosin staining (A) and immunohistochemical staining using anti‐CD68 antibody (B). Scale bar, 100 μm.

CT scan showed no change in the size of the primary tumor and no new lesions. Aiming for surgical complete resection (CR), laparoscopic radical nephrectomy was performed. The nephrectomy specimen showed a 5 cm yellowish brown tumor on the lower pole of the kidney (Figure 3). HE staining revealed a substantial proliferation of tumor cells with clear cytoplasm, leading to the diagnosis of clear cell renal cell carcinoma. Based on pathological findings and history, TNM classification was diagnosed as T1bN0M1. In addition to tumor‐infiltrating lymphocytes, numerous non‐caseating epithelioid cell granulomas were found within the tumor (Figure 4A). The anti‐CD68 monoclonal antibody distinctly highlighted the epithelioid cells (Figure 4B). This was an outbreak of non‐caseating epithelioid cell granuloma in a patient who did not meet the diagnostic criteria for systemic sarcoidosis and was considered SLR due to ICI. In this case, multiple lung metastases appeared at 3 years postoperatively. The department requested a biopsy from a respiratory physician, but the biopsy was not performed due to the decision that it would be difficult to obtain a tissue sample. Clinically, the patient was diagnosed with multiple lung metastases. Comprehensive Genomic Profiling indicated TERT c.‐124c > T, MTOR p.L2427R, RAC1 p.P29S, and VHL p.L158Q as Actionable gene abnormalities. Druggable gene abnormality was noted as MTOR p.L2427R, and he is currently continuing treatment with mTOR inhibitor.

## 3 Discussion

Granulomatous inflammation is a form of chronic inflammation, characterized by the accumulation of syncytial epithelial cells and multinucleated giant cells. Sarcoidosis is a multisystem, systemic disease in which non‐necrotizing granulomas are present in multiple organs including the lung, liver, lymph nodes, spleen, and skin, among others. SLR refers to the formation of non‐caseating epithelioid cell granulomas in patients who do not meet the diagnostic criteria for systemic sarcoidosis. Pathologically, SLR and systemic sarcoidosis are indistinguishable [13]. The etiologies of SLR are not clear. SLR induced by ICI may be related to the modulation of T lymphocytes and antigens derived from destroyed cancer cells [6]. ICI is one of the main treatment modalities for metastatic cancers, including RCC. Among irAEs, the incidence of SLR has been reported to range from 0.2% to 22% [4, 5, 6, 7]. The range in incidence may be due to differences in the drugs used, minor symptoms, and cases misdiagnosed as new metastatic lesions. According to a review by Gkiozos et al., the median onset of SLR after the initiation of ICI is 14 weeks, with many cases reported in melanoma [6]. ICIs that cause SLR include anti‐PD‐1, anti‐PD‐L1, and anti‐CTLA‐4, among which ipilimumab has been reported to be associated with SLR more frequently than other irAEs [5, 6, 7]. This report had a limitation: we cannot prove that the SLR was caused by ICI because there were no pathological findings in the kidney before the administration of ICI. However, since there are few reports of SLR within renal tumors, we consider that it was most likely caused by ICI. In the present case, SLR was observed in the tissue of a kidney removed after four courses of combination therapy with ipilimumab and nivolumab for metastatic renal cell carcinoma. SLR has been reported most often in melanoma and non‐small cell lung cancer, and reports in renal cancer are very rare. To our knowledge, this is the first report of a case of SLR pathologically diagnosed in the kidney in metastatic RCC after ICI treatment. Treatment of SLR basically follows the treatment of systemic sarcoidosis. In most cases, SLR is reported to improve with the discontinuation of ICI treatment and concomitant steroid therapy depending on symptoms [4, 5]. If there are no symptoms or lesions in the central nervous system, eyes, heart, or other organs, follow‐up without discontinuation of ICI may be considered. Chorti et al. reported seven cases of SLR due to ICI, and although ICI treatment was continued after the diagnosis of SLR, SLR remitted or disappeared in six cases and remained unchanged in one case [6, 7]. The appearance of SLR in RCC is rare and very few cases have been reported in the literature [7]. Importantly, SLR can lead to misdiagnosis of newly diagnosed metastatic disease [12]. Tissot C et al. reported a case in which a mediastinal mass and a new micronodular lesion in the lung field were identified on CT, leading to a diagnosis of metastatic disease progression and discontinuation of ipilimumab therapy [14]. We report our experience with a case of ICI‐related SLR in metastatic RCC.

## Ethics Statement

This research was conducted in accordance with the provisions of the Declaration of Helsinki.

## Consent

The authors have nothing to report.

## Conflicts of Interest

The authors declare no conflicts of interest.
